# Determination of hot carrier energy distributions from inversion of ultrafast pump-probe reflectivity measurements

**DOI:** 10.1038/s41467-018-04289-3

**Published:** 2018-05-10

**Authors:** Tal Heilpern, Manoj Manjare, Alexander O. Govorov, Gary P. Wiederrecht, Stephen K. Gray, Hayk Harutyunyan

**Affiliations:** 1 Center for Nanoscale Materials, Argonne National Laboratory, Lemont, IL 60439 USA; 2 Department of Physics, Emory University, Atlanta, GA 30322 USA; 3 Department of Physics and Astronomy, Ohio University, Athens, OH 45701 USA

## Abstract

Developing a fundamental understanding of ultrafast non-thermal processes in metallic nanosystems will lead to applications in photodetection, photochemistry and photonic circuitry. Typically, non-thermal and thermal carrier populations in plasmonic systems are inferred either by making assumptions about the functional form of the initial energy distribution or using indirect sensors like localized plasmon frequency shifts. Here we directly determine non-thermal and thermal distributions and dynamics in thin films by applying a double inversion procedure to optical pump-probe data that relates the reflectivity changes around Fermi energy to the changes in the dielectric function and in the single-electron energy band occupancies. When applied to normal incidence measurements our method uncovers the ultrafast excitation of a non-Fermi-Dirac distribution and its subsequent thermalization dynamics. Furthermore, when applied to the Kretschmann configuration, we show that the excitation of propagating plasmons leads to a broader energy distribution of electrons due to the enhanced Landau damping.

## Abstract (web summary)

The determination of thermal and non-thermal carrier populations in plasmonic systems generally requires assumptions on the types of distributions present. Here, Heilpern et al. directly determine such populations in thin film pump-probe measurements using a double inversion procedure.

## Introduction

The optical generation of non-equilibrium electron distributions in metallic nanostructures and their subsequent dynamics are of fundamental and practical interest due to their potential applications in photovoltaics, photocatalysis, optical switching, and sensing^1–3^. Recent studies, both theoretical and experimental, have generated extensive discussion over the contributions of various physical mechanisms into efficient hot electron generation processes. The term hot electron is often applied to an initial, non-thermal distribution that subsequently relaxes to a thermal distribution and this relaxation dynamics is termed hot electron dynamics even though the initial distribution cannot be described by a temperature.

Complications arising from the computational effort required for full quantum treatments of relatively large nanosystems (>100 nm) have hampered our ability to develop a full fundamental understanding of the new processes that emerge at dimensions comparable to the mean free path of electrons and at characteristic timescales of excited electron relaxation.

**Fig. 1:**
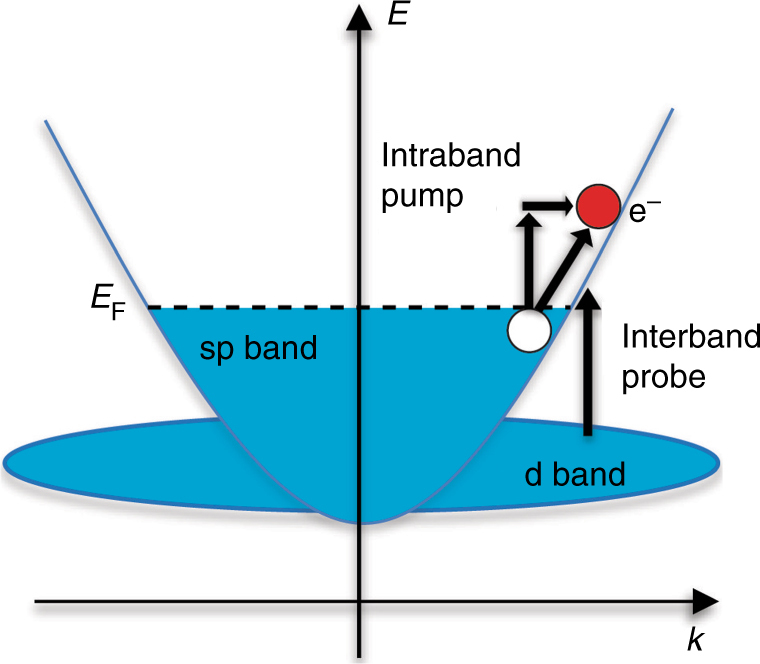
Schematic of the energy band structure and electronic transitions of gold. Arrows indicate an intraband transition in the conduction band initiated by the pump, and an interband transition between the d and conduction bands due to the probe. The creation of an energetic electron (red) and a hole (white) is indicated

Pump-probe optical spectroscopy is commonly used to garner information about ultrafast carrier dynamics in plasmonic systems. Typically, an infrared pump laser pulse excites energetic carriers in the conduction band, whereas a shorter-wavelength probe pulse measures the differential reflectivity (transmission) as a function of time which is associated with the generation and relaxation of excited electrons at interband transition energies, Fig. 1. Plasmon-assisted photoabsorption in noble metals may lead to non-thermal electron distributions via intraband transitions either facilitated by phonon or defect scattering, or a direct diagonal transition due to Landau damping (Fig. 1). The latter is a pure quantum mechanical process where the plasma oscillation energy is transferred to an excited electron–hole pair^4^.

In such hot carrier generation and relaxation, as described in the above paragraph, an interesting picture has emerged from a variety of experimental and theoretical studies^5–15^. The initial photon absorption leads to a non-equilibrium electron energy distribution. Often the change in electron occupancy relative to equilibrium is imagined to be consistent with a double-step-like function^6,7,9^. As time evolves a series of energy redistributions occur where energetic electrons first scatter among themselves and redistribute their energy until a high temperature Fermi–Dirac distribution is reached on the scale of hundreds of femtoseconds. Electron–phonon interactions then lead to a gradual reduction of this electronic temperature on a picosecond time scale. As the electron occupancies change during the various processes described above so does the permittivity and thus reflectivity. Two-temperature models^6,16–19^ that involve time-dependent characteristic electron and phonon temperatures have also proved to be very useful in understanding the relaxation dynamics. For additional discussion of various phenomena underlying the permittivity changes, including nonlinearities, see refs.^8,13^.

In this work we follow the inverse route, by directly extracting the changes in energetic electron–hole distribution from a double inversion of measured pump-probe reflectivity data of 30 nm thin gold film with light incident both normally and in the Kretschmann configuration^20^. This approach does not rely on the assumption of an early time broad double-step energy distribution change or the two-temperature model. Our scheme successfully maps out the dynamics during the ultrafast non-Fermi–Dirac excitation process and the subsequent thermalization by first inverting the relation between the changes in reflectivity and changes in the dielectric function, followed by inverting the relation between the dielectric function and the energy distribution change. Upon excitation of propagating plasmons our calculations hint at increased Landau damping rate due to transverse confinement. The approach can be applied to other materials and structures, given that the unperturbed energy band structure is known, and also that some model connecting the unperturbed dielectric function and unperturbed reflectivity is known. The current model assumes constant band structure and is therefore most applicable to processes that occur before the thermalization of the lattice.

## Results

### Experiments

A 30 nm gold film on top of a glass substrate is considered and pump-probe transient reflectivity measurements are carried out. A 130 fs pump pulse of monochromatic light at *λ*_0_ = 1200 nm (photon energy *ħω*_0_ = 1.0 eV) is followed by a broadband probe pulse in the visible range. In this paper we consider two cases: the pump light incident normally from the air side and a Kretschmann configuration with the pump light incident at the appropriate Kretschmann angle (42.3°) from the glass side. The probe beam is slightly detuned from the Kretschmann angle so that the probe pulse does not excite plasmons and measures only reflectivity. The differential reflectivity, Δ*R*/*R*, is then determined as a function of time *t* and probe frequencies *ω* = 2*πc*/*λ*. The experimental sensitivity is better than a Δ*R*/*R* of 10^−4^, resulting in high quality spectra that are needed for precise results through the double inversion procedure. The skin depth of the Au in this spectral range is ~5 nm, so that all of the optical absorption response of the film can be measured by the Δ*R*/*R* response. Figure 1 shows a schematic of the energy band structure in gold and the intraband and interband transitions corresponding to the excitation of the pump and probe pulses, respectively. For additional experimental details, please see the Methods section.

It is important to verify that the effects discussed herein are not due to other, nonlinear optical processes as can sometimes be the case in plasmonic systems^21^. The analysis of the power dependence of the pump-probe signal verifies that the contribution of multi-photon absorption processes is negligible (see Supplementary Note 9 for detailed analysis of power dependence).

### The double inversion procedure

For a given pump-probe set-up, the reflectivity *R* from the glass-gold structure corresponding to any time *t* and probe frequency *ω* can also be viewed to be a function of the metal’s instantaneous complex dielectric function $\epsilon$ = $\epsilon \prime$ + *i*$\epsilon \prime\prime$. If we denote the unperturbed dielectric function of the metal prior to the pump as $\epsilon_e = \epsilon \prime_{\!\!e} + i\epsilon \prime\prime_{\!\!\!\!e}$, one can write^6^

1 $$\left. {\frac{{{\mathrm{\Delta }}R}}{R} = \frac{{\partial {\kern 1pt} {\mathrm{ln}}{\kern 1pt} R}}{{\partial \epsilon \prime }}} \right|_{\mathrm{e}}{\mathrm{\Delta }}\epsilon \prime + \left. {\frac{{\partial {\kern 1pt} {\mathrm{ln}}{\kern 1pt} R}}{{\partial \epsilon \prime\prime }}} \right|_{\mathrm{e}}{\mathrm{\Delta }}\epsilon \prime\prime ,$$

where the derivatives are taken at the unperturbed dielectric constant values and $\epsilon$ = $\epsilon _e$ + Δ$\epsilon$ has been assumed, which is valid for the relatively small changes that actually occur in such experiments. These derivatives (which depend on *ω*) are obtained numerically (see Methods). We can now use the Kramers–Kronig relation to obtain an integral equation just for $\Delta \epsilon \prime\prime$,

2 $$\frac{{{\mathrm{\Delta }}R}}{R} = \left. {\frac{{\partial {\kern 1pt} {\mathrm{ln}}{\kern 1pt} R}}{{\partial \epsilon \prime\prime }}} \right|_{\mathrm{e}}{\mathrm{\Delta }}\epsilon \prime\prime + \frac{2}{\pi }\left. {\frac{{\partial {\kern 1pt} {\mathrm{ln}}{\kern 1pt} R}}{{\partial \epsilon \prime }}} \right|_{\mathrm{e}}{\mathrm{PV}}\mathop {\int}\limits_0^\infty {{\mathrm{d}}\omega {\prime}\frac{{\omega {\prime}{\mathrm{\Delta }}\epsilon \prime\prime (\omega {\prime})}}{{(\omega {\prime})^2 - \omega ^2}}}$$

where PV stands for Principal Value.

The connection between changes in the dielectric function $\epsilon$ and changes in electron energy distribution is through the energy distribution of the joint density of states (EDJDOS) *D*(*ħω*, *E*)^22,23^ which is a 2-dimensional distribution function counting transitions due to photons of energy *ħω*, which excite electrons to final energy of *E*. Notice that the pump is in the infrared and so its photon energies of 1.03 eV are insufficient to cause interband transitions, however the probe energies assess the gold interband transitions. Thus one has a picture of possibly an initial, broad (±1.03 eV relative to the Fermi energy) initial disturbance in the carrier energy distribution that is subsequently being probed by photons involving primarily interband transitions and so the EDJDOS function needs to involve the interband transitions. This function can be evaluated explicitly for interband transition between the d-band and the s-p conduction band assuming a parabolic structure approximation of the energy bands^24,25^. In gold the two relevant transitions for our probe photon energies are located around the L and X points in the Brillouin zone and we include contributions from both points in our expression for the EDJDOS (see Supplementary Notes 2 and 3). Specifically,

3 $$\epsilon \prime\prime (\omega ) = \epsilon \prime\prime _{{\!\!\!\!\mathrm{intra}}} (\omega ) + \frac{A}{{(\hbar \omega )^2}}\mathop {\int}\limits_{E_{{\mathrm{min}}}}^{E_{{\mathrm{max}}}} {\kern 1pt} D(\hbar \omega ,E)(1 - f(E)){\mathrm{d}}E,$$

where 1 − *f*(*E*) is the probability that an upper band state of energy *E* is empty.

As detailed in the Supplementary Notes 1–6, we determine the parameter *A* by requiring that Eq. (3) approximate the empirical Johnson and Christy^26^ data when *f*(*E*) is taken to be *f*_e_(*E*), the Fermi-Dirac distribution at room temperature. $\epsilon \prime\prime _{{\!\!\!\!\mathrm{intra}}}$ is the intraband contribution to the imaginary part of the dielectric constant which we take to be a Drude form fit to the lower frequency empirical data; for optical frequencies $\epsilon \prime\prime _{{\!\!\!\!\mathrm{intra}}}$ is small relative to the interband contributions. The change Δ*f*(*E*) = *f*(*E*) − *f*_e_(*E*) in the electron energy distribution after the pump is

4 $${\mathrm{\Delta }}\epsilon \prime\prime (\omega ) = - \frac{A}{{(\hbar \omega )^2}}\mathop {\int}\limits_{E_{{\mathrm{min}}}}^{E_{{\mathrm{max}}}} {\kern 1pt} D(\hbar \omega ,E){\mathrm{\Delta }}f(E){\mathrm{d}}E.$$

*D*(*ħω*, *E*) and the integral limits are obtained from explicit expressions^24,25^ using various energies and masses inferred from ref.^27^ (see also Supplementary Notes 2 and 3). Thus, given $\Delta \epsilon \prime\prime$(*ω*) inferred from solution of integral Eq. (2), integral Eq. (4) can be solved to determine Δ*f*(*E*).

We should note that our approach is most readily applicable when reasonable analytical approximations for the relevant energy bands are available. Our detailed analysis (see Supplementary Notes 1–6) to obtain the EDJDOS entering in Eqs. (3) and (4) assumed parabolic approximations for the d and conduction (sp) bands and this is appropriate for noble metals Au and Ag, corresponding to Drude-like plasmonic dynamics in the conduction band and a relatively flat d band. In principle, of course, one could extend our approach beyond these metals with appropriate characterization of the relevant material’s band structure. One could also extend our approach beyond flat films to, for example, metal nanoparticles, but one would have to characterize the reflectance entering into Eqs. (1) and (2) with computational electrodynamics calculations.

### Validation with model data

**Fig. 2:**
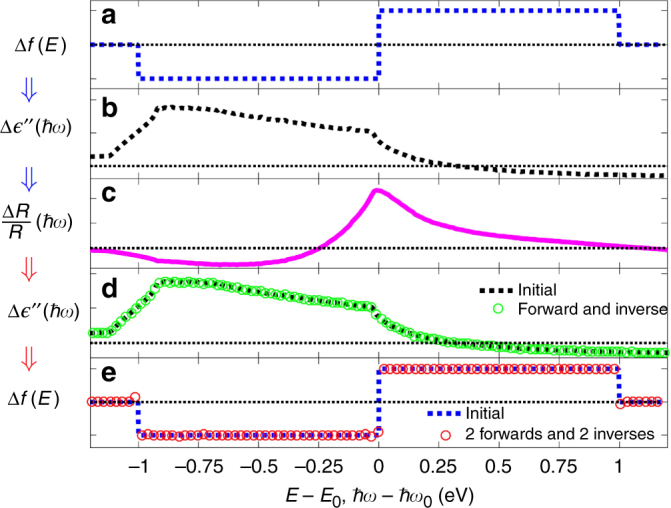
Validation of the double inversion method. **a** A hypothetical change in the electron energy distribution, Δ*f*(*E*). **b** The corresponding $\Delta \epsilon \prime\prime$(*ħω*) due to Δ*f*(*E*). **c** The corresponding differential reflectivity, Δ*R*/*R*(*ħω*) inferred with the perturbed metallic dielectric function. In our procedure we take Δ*R*/*R*(*ħω*) and invert the relevant integral equations to obtain $\Delta \epsilon \prime\prime$(*ħω*), **d**, and Δ*f*(*E*), **e**. The latter agrees well with the original form, **a**. Note that in the upper and lower panels the *x* axis is *E*(eV), where zero refers to the Fermi level, while in the three middle panels the *x* axis is *ħω*, where zero refers to the gap between the d-band and the conduction band of the L-point, which is roughly 2.45 eV

To validate the double inversion procedure we specified a hypothetical electron energy distribution change, Δ*f*(*E*), to be a double-step-like change, Fig. 2a. We used it with Eq. (3) to generate the $\Delta \epsilon \prime\prime$(*ω*) change, Fig. 2b, and used the Kramers-Kronig relation to generate the corresponding Δ$\epsilon \prime$(*ω*). Finally, we used Fresnel calculations to find Δ*R*(*ω*)/*R*(*ω*) = [*R*(*ω*; $\epsilon$ = $\epsilon _e$ + Δ$\epsilon$) − *R*(*ω*; $\epsilon _e$)]/*R*(*ω*; $\epsilon _e$), Fig. 2c. These calculations serve to provide Δ*R*(*ω*)/*R*(*ω*) data, Fig. 2c, for which the electron energy distribution change, Fig. 2a is known. We now apply our first inversion procedure to this reflectivity data, which involves numerically inverting Eq. (2) to obtain $\Delta \epsilon \prime\prime$, Fig. 2d, and then inserting this result into Eq. (3) and numerically inverting it to obtain, Δ*f*(*E*), Fig. 2e. It is clear that to a good approximation the correct energy distribution change, Fig. 2a, is in fact obtained. (The blue points in Fig. 2e are the initial distribution points of Fig. 2a). We now consider cases with experimental data for Δ*R*/*R* where the underlying Δ*f*(*E*) is unknown.

### Inversion of normal incidence data

**Fig. 3:**
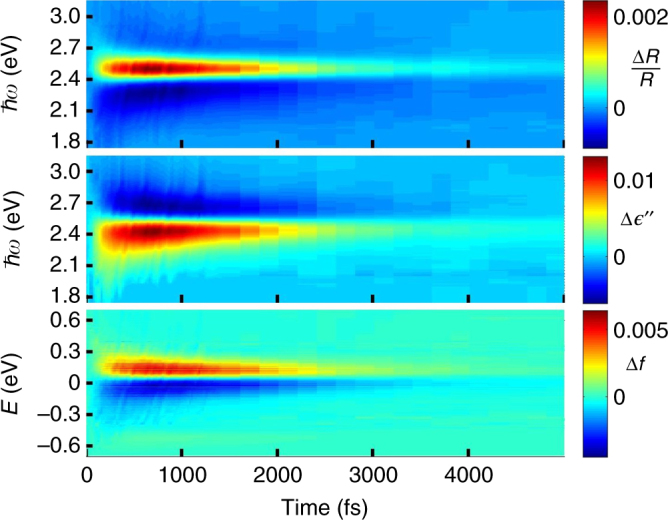
Inversion of experimental pump-probe data. Maps show the changes in reflectivity, dielectric function and electron occupancy changes for the normal 30 nm configuration: **a** Δ*R*/*R*(*ħω*); **b** $\Delta \epsilon \prime\prime$(*ħω*) solved using the integral Eq. (2); **c** Δ*f*(*E*) solved using the second integral Eq. (4)

Figure 3a displays experimental transient (differential) reflectivity measurements of Δ*R*/*R* for a 30 nm gold film in the normal incidence configuration where the pump and probe are incident from the air side and a glass layer is under the gold film. The increasing (red) and decreasing (blue) changes in reflectivity are clearly seen around a probe photon energy *ħω* ≈ 2.4 eV, on a scale of a few picoseconds. Figure 3b presents the map of our inferred $\Delta \epsilon \prime\prime$(*ω*) from (2), where the structure of positive and negative peaks is opposite to the structure of the peaks in the reflectivity. Figure 3c depicts the map of the population changes Δ*f*(*E*) inferred from (4), where now the positive and negative peak structures follow the same order as those in the differential reflectivity.

**Fig. 4:**
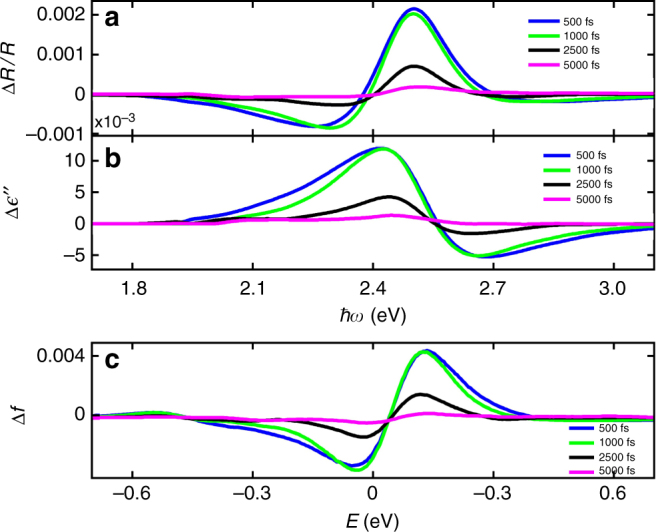
Spectrum cuts of the data and the inverted quantities. Spectrum cuts at times 500, 1000, 2000, 5000 fs for the normal 30 nm configuration: **a** Δ*R*/*R*(*ħω*); **b** $\Delta \epsilon \prime\prime$(*ħω*) solved using the integral Eq. (2); **c** Δ*f*(*E*) solved using the second integral Eq. (4)

Figure 4 presents several spectrum cuts (vertical cuts) of the maps of Fig. 3 at *t* = 500, 1000, 2000, 5000 fs for the normal 30 nm configuration, where Fig. 4a shows plots of the Δ*R*/*R* data, Fig. 4b shows the inferred $\Delta \epsilon \prime\prime$(*ħω*) solved using the integral Eq. (2), and Fig. 4c presents the inferred Δ*f*(*E*) solved using the second integral Eq. (4). It is seen that there is only a minor difference between the curves corresponding to *t* = 500 fs and *t* = 1000 fs, while the changes start to decay after that time. The observed distribution at these relatively late times corresponds to a Fermi–Dirac distribution at an elevated temperature relative to the asymptotic (room) temperature. We find that the maximum electronic temperature in this case is ~1000 K which is within a factor of two of simple estimates based on an extended two-temperature model^10,16^ (see Supplementary Note 8). The origins of this discrepancy should be analyzed further and more sophisticated models may need to be investigated, including more complete Boltzmann equation approaches^28^.

**Fig. 5:**
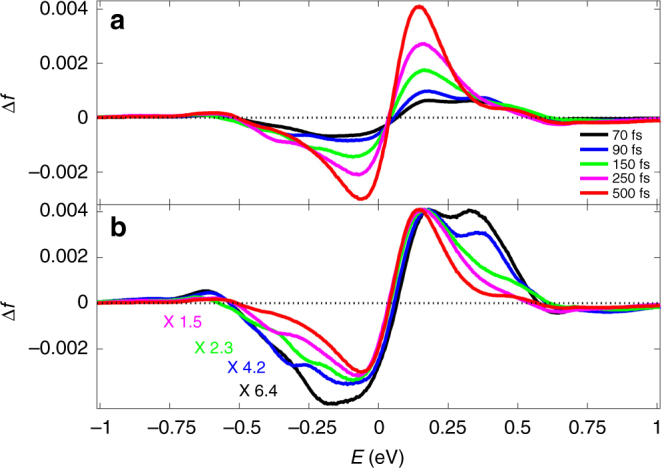
Changes in carrier population for the normal incidence. **a** Spectrum cuts of Δ*f*(*E*), inferred from the second integral Eq. (4), at *t* = 70, 150, 250, 500 fs. The relatively broad initial electron energy distribution change at the earlier time (70 fs) evolves to a narrower and larger magnitude Fermi–Dirac distribution at later time (500 fs). **b** Same as **a** but with multiplication factors in order to better compare the various time functional forms

Figure 5a, b shows electron energy distribution changes for earlier times. The results presented, from *t* = 70 to 500 fs show an early broad electron energy distribution change at 70 fs that is somewhat different from the double-step-like forms often assumed and then the subsequent growth of a much larger magnitude Fermi–Dirac like function at later times. Unfortunately, owing to weak/noisy signals, we could not obtain reliable results for times much <70 fs. At these early times the electron distribution has several interesting characteristics. First it is clearly not a Fermi-Dirac distribution indicating the presence of non-thermalized electron distribution in the system. The fact that it deviates from a double-step-function shape and does not extend all the way to ±1 eV (pump photon energy) relative to the Fermi energy can be attributed to the fact that thermalization process is very fast and therefore the reflectivity measurement at these early times shows some time-averaging. In other words highly excited hot carriers already start decaying within the rise time of our pump pulse. Second, there is a slight asymmetry between the electron and hole distribution which is related to the difference in electron and hole density of states, as will be explained below. From Fig. 5 one sees the hole population (negative energies) extends down to −0.65 eV whereas the excess electron population (positive energies) extends up to 0.75 eV.

There are several physical origins of the permittivity and electron occupancy changes discussed here. The initial 1.0 eV photons on the ultrafast time scale appear to create an equilibrium, Fermi-smeared distribution corresponding to the holes below the Fermi energy and excess electrons above it on a 500–1000 fs time scale followed by a subsequent relaxation to phonons, on a ps time scale similar to many studies, for example, ref. ^6^. There may be some band shifting, possibly due to thermal expansion, that could be responsible for the slight shifting of the apparent Fermi energy or chemical potential, that is, the fact the Δ*f*(*E*) in our figures is not zero at exactly *E* = 0.

### Inversion of Kretschmann configuration data

**Fig. 6:**
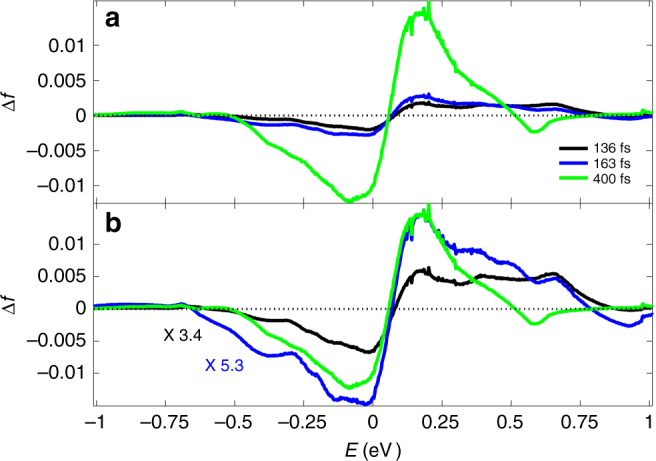
Changes in carrier population for the Kretschmann configuration. **a** Spectrum cuts of Δ*f*(*E*), inferred from the second integral Eq. (4), at *t* = 136, 163, 400 fs. The relatively broad initial electron energy distribution change at the earlier time (136 fs) evolves to a narrower and larger magnitude Fermi–Dirac distribution at later time (400 fs). **b** Same as **a** but with multiplication factors in order to better compare the various time functional forms

Figure 6 displays results of applying the double inversion scheme to reflectivity data measured in the Kretschmann configuration. The Kretschmann energy distribution changes are somewhat wider than those of the normal incidence configuration in Fig. 5, although still it should be noted that they are narrower than the naive expectation, as in Fig. 2a. Figure 6 shows that the early time Kretschmann case hole population extends down to −0.75 eV and the excess electron population extends further up to 1 eV, which can be contrasted with the normal incidence values of −0.65 and 0.75 eV, indicating the broader extent of the early time electron disturbance and greater asymmetry present in the Kretschmann case.

We should note that the slight negative dips in some of the Δ*f* cases of Figs. 5 and 6 for positive energies are likely artifacts due to imperfections in the experimental data and our double inversion procedure. In particular, the effects of data truncation and extrapolation outside the available frequency range, important considerations for the Kramers-Kronig relations, are discussed in Supplementary Note 7.

The somewhat wider distributions in the Kretschmann case (Fig. 6) are likely due to Landau damping of the plasmon^29^ which cannot occur in the case of normal incidence because no plasmons are excited. The overall narrower and somewhat non-symmetric shape of the early time non-Fermi energy distribution change functions relative to naive expectations is consistent with quantum mechanical calculations for the intraband hot electron generation^3,30^ and represents a key result of our paper. Narrower distributions are expected because of two reasons. First, high-energy electrons are created in the processes with non-conservation of linear momentum and, therefore, the probability to create electrons with high excitation energies is smaller than that for low-energy electrons; thus, the distribution tends to be closer to the Fermi level. Second, e–e relaxation times for high energy electrons are shorter and, therefore, the number of such energetic electrons should be smaller. The asymmetry of excited electrons and holes is also well expected^3,30^. Since the density of states depends on energy, the calculated electrons tend to have a wider energy distribution (that is, more distributed between *E*_F_ and *E*_F_ + *ħω*_0_) as compared to the calculated holes^30^; such character of the distributions was found in quantum calculations. It should be also be noted that Landau damping and the resulting direct intraband transitions (diagonal arrow in Fig. 1) typically need much smaller real space confinement of fields and respective wavevectors to become resonant. However, the propagating SPPs that decay evanescently normal to the gold surface will inevitably have some wavevector components that will fall in this range. This electron decay channel can be thought of as one of the most fundamental loss mechanisms for propagating plasmons in metals. To the best of our knowledge this is the first direct observation of ultrafast Landau damping in metal plasmonic systems.

It is important to note that some of the features of the early time energy distribution changes we have inferred from the experimental results do also emerge from certain other theoretical analyses and simulations. A master equation approach^31^ to non-thermal electron relaxation after optical excitation in small gold and silver nanoparticles also showed significantly greater and broader responses on plasmon resonance as we have found. The Boltzmann equation simulations of ref. ^28^ show an evolution of the electron distribution for a silver film that is consistent with a somewhat narrower distribution resulting after a short (20 fs) pulse. Interestingly, an extended two-temperature model^9^ of non-thermal electron dynamics in gold films, which correlated well with experiment, also showed early time electron energy distributions with structures closer to the Fermi energy.

## Discussion

A double inversion scheme enables direct determination of nonthermal and thermal carrier distributions from pump probe reflectivity data. It does not rely on assumptions such as an early time broad double-step occupancy change and the two-temperature model and its extensions for subsequent energy transfer. We showed that this approach allows one to take measured transient reflectivity data and determine the evolution of the carrier energy distribution from early to late times. Applying the approach to pump-probe data for a 30 nm thin gold film showed the dynamics of the fast non Fermi–Dirac excitation process and the subsequent thermalization dynamics of this hot electron distribution. Both normal incidence and Kretschmann reflection configurations were analyzed, with the system response being significantly higher, and with more energetic electrons generated in the Kretschmann case. Thus, our results indicate that plasmon-assisted electromagnetic field confinement may lead to a modification of absorption properties and ultrafast carrier dynamic in materials. Our approach to determining the energy distribution of hot carriers is extendable to other plasmonic nanostructures and illumination geometries, enabling new opportunities in understanding and utilizing hot carrier generation processes.

## Methods

### Experimental methods

Time-resolved pump-probe spectroscopy was performed using an amplified Ti:sapphire laser system equipped with an optical parametric amplifier (OPA). This system was used to generate tunable near-infrared pump pulses to excite intraband transitions as well as a variably delayed continuum probe extending from 350 to 1000 nm. The pulses are 130 fs in duration and the excitation of the samples occurs at 2.5 kHz. The specific experiments discussed herein correspond to a laser power 500 μW with incident pump pulse energy on the sample of 200 nJ per pulse, focused to a 200-μm-diameter spot. The fluence was correspondingly 640 μJ cm^−2^. The reflected continuum probe was collected and dispersed into a spectrograph that used either a visible (Si) or near-infrared (InGaAs) array detector.

### Computational methods

The derivatives entering into Eqs. (1) and (2) were obtained numerically using the standard Fresnel equations and assuming the unperturbed metal dielectric constant to base changes upon is given by the empirically determined Johnson-Christy data^26^.

Each of the two integral equations associated with the double inversion scheme, Eq. (2) for finding $\Delta \epsilon \prime\prime$(*ω*), and Eq. (4) for finding Δ*f*(*E*), were solved with standard discretization and regularization techniques. In particular, the regularizations of the relevant pseudo inverse matrices were carried out with the L-curve method^32^. See the Supplementary Note 5 for a detailed discussion.

### Data availability

The data that support the findings of this study are available from the corresponding authors on reasonable request.

## Electronic supplementary material

Supplementary Information

## References

[1] Brongersma ML, Halas NJ, Nordlander P (2015). Plasmon-induced hot carrier science and technology. Nat. Nanotechnol..

[2] Gieseking, R. L., Ratner, M. A. & Schatz, G. C. In *Frontiers of Plasmon Enhanced Spectroscopy* Vol. 1 (eds Ozaki, Y. et al.) 1–22 (ACS Publications, 2016).

[3] Hartland GV, Besteiro L, Johns P, Govorov AO (2017). What’s so hot about electrons in metal nanoparticles?. ACS Energy Lett..

[4] Khurgin J, Tsai WY, Tsai DP, Sun G (2017). Landau damping and limit to field confinement and enhancement in plasmonic dimers. ACS Photonics.

[5] Fann W, Storz R, Tom H, Bokor J (1992). Direct measurement of nonequilibrium electron-energy distributions in subpicosecond laser-heated gold films. Phys. Rev. Lett..

[6] Sun CK, Vallée F, Acioli LH, Ippen EP, Fujimoto JG (1994). Femtosecond-tunable measurement of electron thermalization in gold. Phys. Rev. B.

[7] del Fatti N (2000). Nonequilibrium electron dynamics in noble metals. Phys. Rev. B.

[8] Voisin C, Fatti N, Christofilos D, Vallee F (2001). Ultrafast electron dynamics and optical nonlinearities in metal nanoparticles. J. Phys. Chem. B..

[9] Della Valle G, Conforti M, Longhi S, Cerullo G, Brida D (2012). Real-time optical mapping of the dynamics of nonthermal electrons in thin gold films. Phys. Rev. B.

[10] Conforti M, Della Valle G (2012). Derivation of third-order nonlinear susceptibility of thin metal films as a delayed optical response. Phys. Rev. B.

[11] Marini A (2013). Ultrafast nonlinear dynamics of surface plasmon polaritons in gold nanowires due to the intrinsic nonlinearity of metals. New. J. Phys..

[12] Kornbluth M, Nitzan A, Seideman T (2013). Light-induced electronic non-equilibrium in plasmonic particles. J. Chem. Phys..

[13] Stoll T, Maioli P, Crut A, Del Fatti N, Vallee F (2014). Advances in femto-nano-optics: ultrafast nonlinearity of metal nanoparticles. Eur. Phys. J. B.

[14] Harutyunyan H (2015). Anomalous ultrafast dynamics of hot plasmonic electrons in nanostructures with hot spots. Nat. Nanotechnol..

[15] Sykes ME (2017). Enhanced generation and anisotropic coulomb scattering of hot electrons in an ultra-broadband plasmonic nanopatch metasurface. Nat. Commun..

[16] Carpene E (2006). Ultrafast laser irradiation of metals: Beyond the two-temperature model. Phys. Rev. B.

[17] Kaganov M, Lifshitz I, Tanatarov L (1957). Relaxation between electrons and the crystalline lattice. Sov. Phys. JETP.

[18] Allen PB (1987). Theory of thermal relaxation of electrons in metals. Phys. Rev. Lett..

[19] Sadasivam S, Chan MK, Darancet P (2017). Theory of thermal relaxation of electrons in semiconductors. Phys. Rev. Lett..

[20] Kretschmann E (1971). Die bestimmung optischer konstanten von metallen durch anregung von oberflächenplasmaschwingungen. Z. für Phys. A Hadrons Nucl..

[21] Mejard R (2016). Energy-resolved hot-carrier relaxation dynamics in monocrystalline plasmonic nanoantennas. ACS Photonics.

[22] Koyama RY, Smith NV (1970). Photoemission properties of simple metals. Phys. Rev. B.

[23] Smith NV (1971). Photoelectron energy spectra and the band structures of the noble metals. Phys. Rev. B.

[24] Rosei R, Antonangeli F, Grassano U (1973). D bands position and width in gold from very low temperature thermomodulation measurements. Surf. Sci..

[25] Guerrisi M, Rosei R, Winsemius P (1975). Splitting of the interband absorption edge in Au. Phys. Rev. B.

[26] Johnson PB, Christy RW (1972). Optical constants of the noble metals. Phys. Rev. B.

[27] Christensen NE, Seraphin B (1971). Relativistic band calculation and the optical properties of gold. Phys. Rev. B.

[28] Pietanza I, Longo S, Capitelli M (2007). Non-equilbrium electron and phonon dynamics in meetals under femtosecond laser pulses. Eur. Phys. J. D.

[29] Khurgin JB (2015). Ultimate limit of field confinement by surface plasmon polaritons. Faraday Discuss..

[30] Besteiro LV, Kong XT, Wang Z, Harland G, Govorov AO (2017). Understanding hot-electron generation and plasmon relaxation in metal crystals: Quantum and classical mechanics. ACS Photonics.

[31] Saavedra J, Asenjo-Garcia A, Garcia de Abajo F (2016). Hot-electron dynamics and thermalization in small metallic nanoparticles. ACS Photonics.

[32] Hansen PC (1992). Analysis of discrete ill-posed problems by means of the l-curve. SIAM Rev..

