# Supplementary material for: Determination of hot carrier energy distributions from inversion of ultrafast pump-probe reflectivity measurements
Source: Nat Commun. 2018 May 10;9:1853. doi: 10.1038/s41467-018-04289-3 (PMC5945638; doi:10.1038/s41467-018-04289-3)
Supplement: Supplementary file 1 — Supplementary Information [file 41467_2018_4289_MOESM1_ESM.pdf]

Determination of hot carrier energy distributions from inversion of ultrafast pump-probe reflectivity measurements - Supporting Information

Tal Heilpern[1], Manoj Manjare[2], Alexander O. Govorov[3],
Gary P. Wiederrecht[1], Stephen K. Gray[1], Hayk Harutyunyan[2]
[1] *Center for Nanoscale Materials, Argonne National Laboratory, Lemont, IL 60439, USA,*
[2] *Department of Physics, Emory University, Atlanta, GA 30322-2430, USA,*
[3] *Department of Physics and Astronomy, Ohio University, Athens, Ohio 45701, USA*

CONTENTS

Supplementary Note 1. Solution of the first integral equation 2

Supplementary Note 2. Calculation of $D(\hbar\omega, E)$ 3

Supplementary Note 3. Band structure parameterizations 4

Supplementary Note 4. Solution of the second integral equation 4

Supplementary Note 5. Regularization of the pseudo inverse using the L-curve method 5

Supplementary Note 6. Numerical details 6

Supplementary Note 7. Effect of data truncation on the double inversion 6

Supplementary Note 8. Extended two-temperature model 7

Supplementary Note 9. Power dependence 8

Supplementary References 9

SUPPLEMENTARY NOTE 1. SOLUTION OF THE FIRST INTEGRAL EQUATION

The first integral equation of our double inversion procedure (Eq. (2) in the main text),

$$\frac{\Delta R}{R} = \left.\frac{\partial \ln R}{\partial \epsilon''}\right|_{\mathrm{e}} \Delta\epsilon'' + \frac{2}{\pi}\left.\frac{\partial \ln R}{\partial \epsilon'}\right|_{\mathrm{e}} \mathrm{PV}\int_0^\infty d\omega' \frac{\omega' \Delta\epsilon''(\omega')}{(\omega')^2 - \omega^2}, \tag{1}$$

can be solved by assuming that $\Delta\epsilon''(\omega)$ is composed from a sum of square pulse functions $\Delta\epsilon''(\omega) = \sum_{\mathrm{j}} a_{\mathrm{j}} P_{\mathrm{j}}(\omega)$, where $a_{\mathrm{j}} = \mathbf{a}_{\mathrm{j}}$ are the components of the unknown solution vector, and each pulse function $P_{\mathrm{j}}(\omega)$ is localized around ω_{j}, i.e.,

$$P_{\mathrm{j}}(\omega) = \begin{cases} 1, & \omega_{\mathrm{j}} - \Delta\omega/2 < \omega < \omega_{\mathrm{j}} + \Delta\omega/2, \\ 0, & \text{otherwise.} \end{cases} \tag{2}$$

Substituting this sum in Eq.(2) in the main text and enforcing the equation on each data point at ω_i leads to

$$\frac{\Delta R}{R}(\omega_{\mathrm{i}}) = \frac{\partial \ln R}{\partial \epsilon'}(\omega_{\mathrm{i}}) \sum_{\mathrm{j}} a_{\mathrm{j}} \mathrm{P.V}\frac{2}{\pi} \int_{\omega_{\mathrm{j}}-\Delta\omega/2}^{\omega_{\mathrm{j}}+\Delta\omega/2} d\omega' \frac{\omega'}{(\omega')^2 - (\omega_{\mathrm{i}})^2} + \frac{\partial \ln R}{\partial \epsilon''} \sum_{\mathrm{j}} a_{\mathrm{j}} P_{\mathrm{j}}(\omega_{\mathrm{j}}) \tag{3}$$

Finally this equation can be written in a matrix form by defining the data vector $(\mathbf{r})$ which elements are $r_{\mathrm{j}} = \frac{\Delta R}{R}(\omega_{\mathrm{j}})$, the solution coefficients vector $\mathbf{a}$ which elements are a_{j}, solving the integral and noting that $P_{\mathrm{j}}(\omega_i) = \delta_{\mathrm{ij}}$, giving the matrix equation

$$\mathbf{r} = M\mathbf{a}, \qquad \text{where} \tag{4}$$

$$M_{\mathrm{ij}} = \frac{\partial \ln R}{\partial \epsilon''}(\omega_{\mathrm{i}})\delta_{\mathrm{ij}} + \frac{\partial \ln R}{\partial \epsilon'}(\omega_{\mathrm{i}})\frac{2}{\pi}(\frac{-1}{2})\left(\ln(\omega_{\mathrm{i}}^2 - (\omega_{\mathrm{j}} + \frac{\Delta\omega}{2})^2) - \ln(\omega_{\mathrm{i}}^2 - (\omega_{\mathrm{j}} - \frac{\Delta\omega}{2})^2)\right)$$
$$= \frac{\partial \ln R}{\partial \epsilon''}(\omega_{\mathrm{i}})\delta_{\mathrm{ij}} + \frac{\partial \ln R}{\partial \epsilon'}(\omega_{\mathrm{i}})\frac{2}{\pi}(\frac{-1}{2})\left(\ln\frac{\omega_{\mathrm{i}}^2 - (\omega_{\mathrm{j}} + \frac{\Delta\omega}{2})^2}{\omega_{\mathrm{i}}^2 - (\omega_{\mathrm{j}} - \frac{\Delta\omega}{2})^2}\right) \tag{5}$$

A simple inverse of the matrix M might not exist, hence a regularized pseudo inverse should be applied as explained in Supplementary Note 4.

SUPPLEMENTARY NOTE 2. CALCULATION OF $D(\hbar\omega, E)$

Following Rosei and co-workers [1, 2] we calculate $D(\hbar\omega, E)$, the energy distribution of the joint density of states (EDJDOS) with respect to the energy of the final state associated with transitions from a lower (l) to upper (u) band,

$$D(\hbar\omega, E) = \int d^3k\delta(E - E_{\rm u})\delta(E_{\rm u} - E_{\rm l} - \hbar\omega) \quad . \tag{6}$$

The lower and upper bands are assumed to be parabolic in character near the relevant transition points,

$$E_{\rm u} = X_{\rm u} + \frac{\hbar^2}{2m_{\rm u\perp}}k_\perp^2 - \frac{\hbar^2}{2m_{\rm u\parallel}}k_\parallel^2 \tag{7}$$

$$E_{\rm l} = X_{\rm l} - \frac{\hbar^2}{2m_{\rm l\perp}}k_\perp^2 - \frac{\hbar^2}{2m_{\rm l\parallel}}k_\parallel^2 \tag{8}$$

with the convention that all the masses are positive. Use of these expressions with a line integral equivalent of Supplementary Equation (6) [1] can be shown to lead to

$$D(\hbar\omega, E) = \frac{1}{8\pi^2\hbar^2}\Big(\frac{m_{\rm l\perp}m_{\rm u\parallel}m_{\rm l\parallel}m_{\rm u\parallel}}{m_{\rm l\perp}m_{\rm u\parallel} + m_{\rm l\parallel}m_{\rm u\perp}}\Big)^{1/2}\Big(\frac{\hbar^2}{2m_{\rm u\perp}}(\hbar\omega - E + X_{\rm l}) - \frac{\hbar^2}{2m_{\rm l\perp}}(E - X_{\rm u})\Big)^{-1/2} \quad . \tag{9}$$

It is important to note that implicit in Supplementary Equation (9) are the two conditions implied by Supplementary Equation (6), namely that

$$E = E_{\rm u}(k_\perp^2,\ \ k_\parallel^2) \quad . \tag{10}$$

and

$$\hbar\omega = E_{\rm u}(k_\perp^2,\ \ k_\parallel^2) - E_{\rm l}(k_\perp^2,\ \ k_\parallel^2) \tag{11}$$

For given E and ω these two equations can readily be solved for $k_\perp^2$ and $k_\parallel^2$ and if either of these is negative then one must set $D = 0$. (Note that simply checking that the argument of the square root in Supplementary Equation (9) is greater than zero is not sufficient.)

For gold at the frequencies of interest there are two points in the Brillouin zone of relevance, the L and X points. As such there are two terms of the form Supplementary Equation (9), each with different lower and upper band parameterizations, and the full EDJDOS is written

$$D(\hbar\omega, E) = D_{\rm L}(\hbar\omega, E) + a_{\rm L/X}D_{\rm X}(\hbar\omega, E), \tag{12}$$

where $a_{\rm L/X}$ is the ratio between the different L and X contributions.

The connection between ϵ'' and the room temperature $T_r = 298$K Fermi-Dirac distribution $f(E) = 1/(e^{E/kT_{\rm r}} + 1)$ is

$$\epsilon''(\omega) = \epsilon_{\rm intra}''(\omega) + \frac{A}{(\hbar\omega)^2}\int_{E_{\rm min}}^{E_{\rm max}} D(\hbar\omega, E)(1 - f(E))dE \quad . \tag{13}$$

This integral can be performed numerically over a suitably broad range of energies (e.g., $E_{\rm min}$ = - 5 eV and $E_{\rm max}$ = 5 eV) and keeping in mind the conditions on each contributing EDJDOS factor as discussed below Supplementary Equation (9). (Alternatively one can use these conditions to determine the integration range.) It is possible for the argument of the square root in Supplementary Equation (9) to become very small for energies near where the conditions become unsatisfied and we have found that simply setting a non-zero cut-off minimum value for the argument is sufficient for obtaining good results. The parameters $a_{\rm L/X}$ and A are related to electronic transition moments and are not readily available. However, by evaluating Supplementary Equation (13) as outlined above for varying $a_{\rm L/X}$ and A, one can obtain a fit with the empirical dielectric function of Johnson and Christy [3], and the result is presented in Supplementary Figure 1. The value of $a_{\rm L/X}$ in Supplementary Equation (12) was fit to be 0.4 which is close to the values found by Guerrisi et al., [2] which were 0.32 and 0.37, and the value of A in Supplementary Equation (13) was found to be 10 atomic units (hartree^3 a$_0^3$).

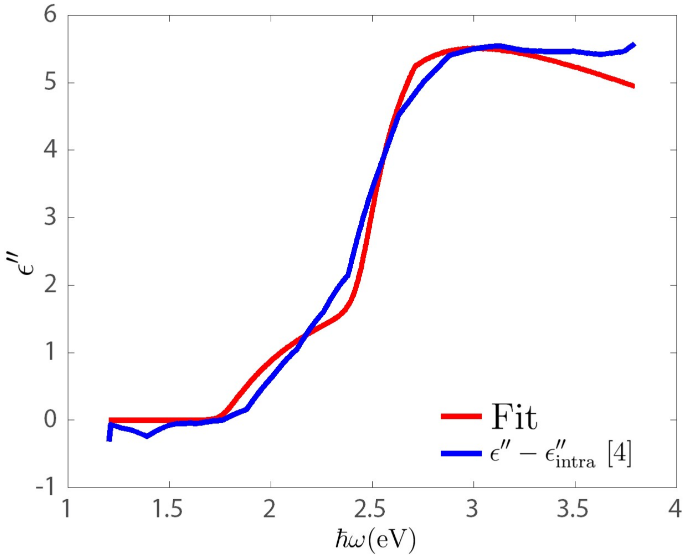

SUPPLEMENTARY FIGURE 1: Fit of ϵ'' using the fit of the D function (red), and comparison to the $\epsilon'' - \epsilon_{\mathrm{intra}}''(\omega)$ measured by Johnson and Christy [3] in blue.

SUPPLEMENTARY NOTE 3. BAND STRUCTURE PARAMETERIZATIONS

The band energies and electron masses were inferred from Christensen and Seraphin. [4]

	X_{u}	$m_{\mathrm{u}\parallel}$	$m_{\mathrm{u}\perp}$	X_{l}	$m_{\mathrm{l}\parallel}$	$m_{\mathrm{l}\perp}$
L point	-0.7167 eV	0.2535 m_{e}	0.2219 m_{e}	-2.2944 eV	0.8113 m_{e}	0.8690 m_{e}
X point	1.5242 eV	0.1206 m_{e}	0.2410 m_{e}	-1.5800 eV	1.0282 m_{e}	0.7017 m_{e}

SUPPLEMENTARY TABLE I: Band structure parameters. Parameters used in band structure calculations for L and X points.

Note that the energy positions the bands, X_{u} and X_{l} above, are given relative to the Fermi level (7.208 eV) as the zero of energy.

SUPPLEMENTARY NOTE 4. SOLUTION OF THE SECOND INTEGRAL EQUATION

Here we aim to solve the second and final integral equation of our double inversion procedure (Eq. (4) of the main text),

$$\Delta\epsilon''(\omega) = -\frac{A}{(\hbar\omega)^2}\int_{E_{\mathrm{min}}}^{E_{\mathrm{max}}} D(\hbar\omega, E)\Delta f(E)dE \quad . \tag{14}$$

Next, as in the first integral equation, we express the solution function Δf as a sum of non-overlapping square pulse functions, i.e., $\Delta f = \sum_{\mathrm{j}} b_{\mathrm{j}} P_{\mathrm{j}}(E)$, where:

$$P_j(E) = \begin{cases} 1, & E_j - \Delta E/2 < E < E_{\mathrm{j}} + \Delta E/2, \\ 0, & \text{otherwise.} \end{cases} , \tag{15}$$

and substituting back inside the integral equation gives:

$$\Delta\epsilon''(\hbar\omega) = \sum_{\mathrm{j}} b_{\mathrm{j}} \int_{\max(E_{\mathrm{j}}-\Delta E/2, E_{\mathrm{min}})}^{\min(E_{\mathrm{j}}+\Delta E/2, E_{\mathrm{max}})} -\frac{A}{(\hbar\omega)^2} D(\hbar\omega, E)dE \tag{16}$$

Applying this equation for each data point $\Delta\epsilon''(\hbar\omega_\mathrm{i})$ gives a set of equation written in matrix form:

$$\mathbf{\Delta}\epsilon'' = N\mathbf{b} \tag{17}$$

where $\mathbf{\Delta}\epsilon''$ is the vector of data points (result of the first inversion of the data), $\mathbf{b}$ is the vector of unknown coefficients which needs to be solved, and where

$$N_\mathrm{ij} = \int\limits_{\max(E_\mathrm{j}-\Delta E/2, E_\mathrm{min})}^{\min(E_\mathrm{j}+\Delta E/2, E_\mathrm{max})} -\frac{A}{(\hbar\omega)^2} D(\hbar\omega_\mathrm{i}, E)dE \tag{18}$$

The naive solution is $\mathbf{c} = N^{-1}\mathbf{J}$, however the matrix N is highly singular and an inverse does not exists. Therefore the solution is achieved using a pseudo inverse which is regularized using the L-Curve method.

SUPPLEMENTARY NOTE 5. REGULARIZATION OF THE PSEUDO INVERSE USING THE L-CURVE METHOD

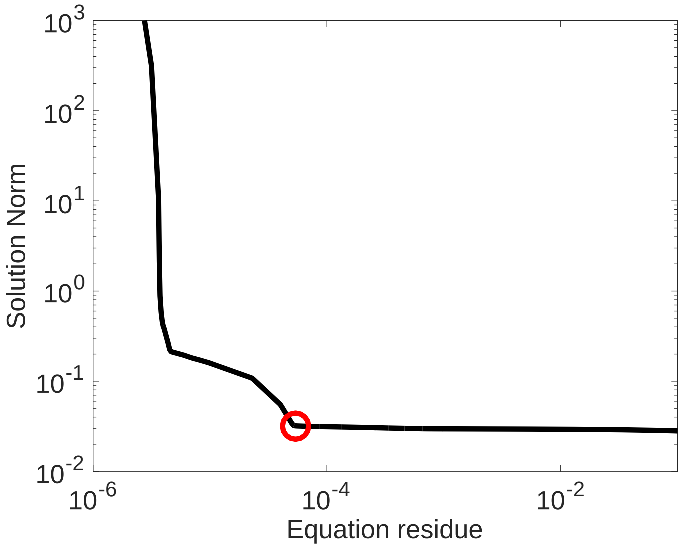

SUPPLEMENTARY FIGURE 2: Example of the L curve used for regularization of the second integral equation. Increasing the number of singular values used in the inversion traces a curve from the down right. Upon arriving on the knee point (marked here with red circle), which correspond in this case for using 507 singular values, the optimal inversion is reached.

Here we briefly summarize how to solve and regularize a matrix equation of the form $\mathbf{r} = M\mathbf{a}$ in cases where the full inverse of M does not exist, using the L-curve method [5]. Obviously, other regularization techniques might be applied. The matrix M is decomposed using the singular value decomposition as

$$M = \sum_\mathrm{j} \sigma_\mathrm{j}\mathbf{u}_\mathrm{j}\mathbf{v}^\dagger \tag{19}$$

where $\mathbf{u}, \mathbf{v}$ are orthonormal vector bases spanning the vector spaces with appropriate dimensions according to the dimension of M, $\{\sigma_\mathrm{j}\}$ is a set of non-increasing real singular values. Note that the decomposition is a sum of outer products of column vector with a row vector. Next, the pseudo inverse of M is written as

$$M^{-I} = \sum_\mathrm{j}^{N_\mathrm{j}} \frac{1}{\sigma_\mathrm{j}}\mathbf{v}_\mathrm{j}\mathbf{u}^\dagger \tag{20}$$

The task is now to the determine the number N_j of meaningful singular values σ_j to be added, since small singular values are attributed to error in the model and may also be below the noise level. The number N_j is therefore data-dependent and needs to be carefully chosen for each specific problem.

In the method of the L-Curve (Supplementary Figure 2), one calculates the solution with a specific number of singular values, and then plots the solution norm (y axis) versus the residue of re-inserting the solution back into the original matrix equation (x axis). Written more simply, given a solution $a^{N_{\rm j}}$ the L-curve is the set of points $\|\mathbf{r} - M\mathbf{a}^{\mathbf{N}_{\rm j}}\|, \|\mathbf{a}^{\mathbf{N}_{\rm j}}\|$. Increasing the number of singular values used in the pseudo inverse, the residue of the matrix equation becomes smaller, since the solution is improving, and therefore the curve will show a horizontal approaching left behavior (reduced residue). After a certain point the singular values $\sigma_{\rm j}$ are too small and describe error in the model and/or values below the noise level. Therefore the inverse term $\frac{1}{\sigma_{\rm j}}$ will result in exploding norm of the solution almost without improvement of the residue (i.e., the solution does not become more exact), and hence the curve will exhibits a vertical leg.

SUPPLEMENTARY NOTE 6. NUMERICAL DETAILS

In the normal incidence configuration, for each time instant we had 532 data points, plus 100 points of extrapolation for each side, summing to 732 points. In the first inversion the L-Curve method yield the result that all singular values should be used, however in the second inversion the elbow was reached at 385 singular values. For the Kretschmann configuration we had respectively 540 data points plus 100 points of extrapolation from each side, summing to 740 points. Out of that, 405 singular values were determined as the optimal number for the second inversion.

SUPPLEMENTARY NOTE 7. EFFECT OF DATA TRUNCATION ON THE DOUBLE INVERSION

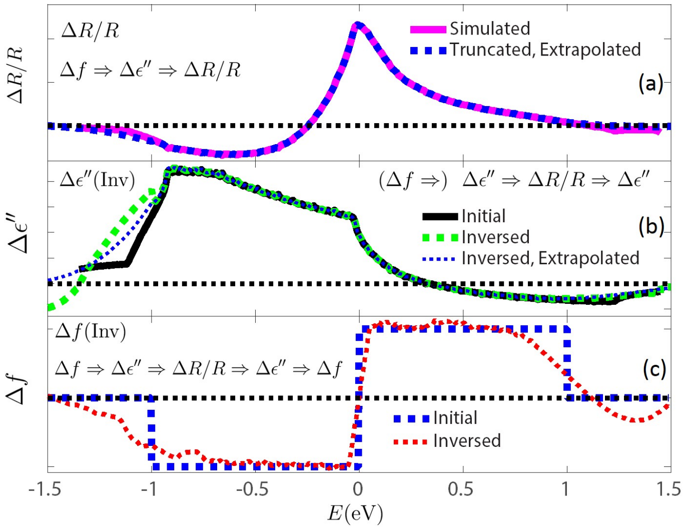

SUPPLEMENTARY FIGURE 3: Example of the extrapolation of the data at the edges, using simulated data which has been synthetically truncated, extrapolated and inverted. (a) upper panel shows a simulated data as in Fig. 2(c) in the main text, which has been truncated at ±1eV above/below Fermi level, and then extrapolated to mimic extrapolation of unkonwn data ; (b) inverted curve is shown together with the original curve used for calculation, and with an truncated and extrapolated curve which avoids discontinuities.; (c) inverted curve along with original Δf

The use of the Kramers-Kronig relation to implicitly account for the real part of the dielectric function changes requires that the imaginary part, $\Delta\epsilon''(\omega)$ be known for a sufficiently broad range of ω values such that it is effectively zero outside this range. This means, in turn, that the experimental $\Delta R/R(\omega)$ data must also be known over such a sufficiently broad range of ω. While this is generally the case for most of the experimental data we have analyzed, there can be situations, particularly when one is examining the very short-time dynamics with very small $\Delta R/R$ responses that are somewhat noisy that some sort of extrapolation of the results outside the known region is necessary. This can be a subtle issue and care must be taken to ensure that the results so-obtained are reliable.

Here we demonstrate the effect of the extrapolation of the data at the edges, using simulated data which has been synthetically truncated above/below Fermi level, then extrapolated with smooth decay to mimic the extrapolation done on incomplete data, and finally inverted. At the second step, the result is checked and if there is large non-physical

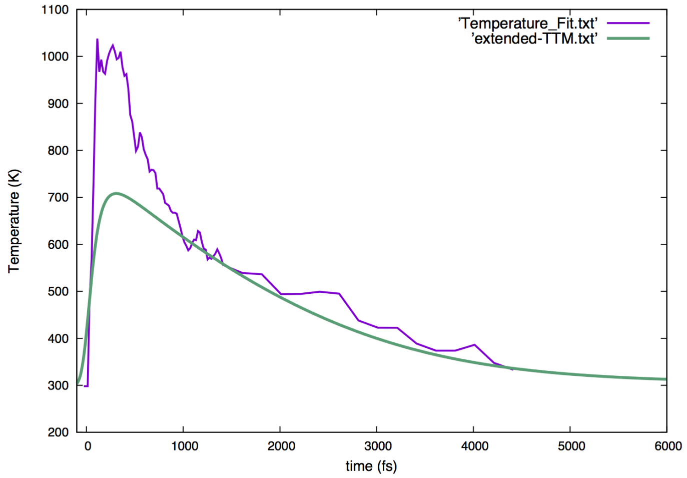

SUPPLEMENTARY FIGURE 4: An extended two-temperature model result for ΔT_e (green curve) is compared with the $T_{\rm e}$ inferred from our present approach (purple curve).

discontinuity/behavior, the result is again truncated and extrapolated at the same points of the original truncation. It should be noted that since the original simulated data is created starting from the box-like step functions, which have strong discontinuity, then the simulated data also has strong discontinuities, and the extrapolation done is therefore very sensitive to cases where the truncation and extrapolation is done before the discontinuity point, before the slope has changed. In such cases the extrapolation will miss the correct decay, however, we show here that the inversion procedure still yields reasonable results. Supplementary Figure 3(a) shows the simulated data in purple, and the truncated and extrapolated curve in dashed blue. Next, Supplementary Figure 3(b) shows the inversion result in dashed green, along the original theoretic calculated curve in black. When extrapolating the result from the points of truncation, we get better agreement with the theoretical original curve. Supplementary Figure 3(c) shows the final result in dotted red along the box-like functions used to simulate the data in dashed blue. Overall we get a fine agreement with the theoretical boxes.

SUPPLEMENTARY NOTE 8. EXTENDED TWO-TEMPERATURE MODEL

Extended two-temperature models (TTMs) [6–9] can incorporate some aspects of the early-time non-thermal energy density, $N(t)$. We use the following relatively simple extended TTM [9]:

$$\frac{\partial N(t)}{\partial t} = -(a+b)N(t) + P(t) \tag{21}$$

$$C_{\rm e}(t)\frac{\partial T_{\rm e}(t)}{\partial t} = -g[T_{\rm e}(t) - T_{\rm l}(t)] + aN(t) \tag{22}$$

$$C_{\rm l}\frac{\partial T_{\rm l}(t)}{\partial t} = g[T_{\rm e}(t) - T_{\rm l}(t)] + bN(t) \tag{23}$$

with $C_{\rm e}(t) = \gamma T_{\rm e}(t)$, and where $T_{\rm e}(t)$ is the thermalized electron temperature and $T_{\rm l}(t)$ is the thermalized phonon temperature. The rate of absorbed energy density (associated with the growth of non-thermal electrons) is given by $P(t) = AI(t)/\alpha$ where A is the absorbance, α is the absorption coefficient , and $I(t)$ is the laser pulse intensity associated with the pump of the form $B\exp[-4ln(2)(t/t_{pulse})^2]$. The above equations represent a simple set of three coupled first-order differential equations in time that can be readily solved numerically.

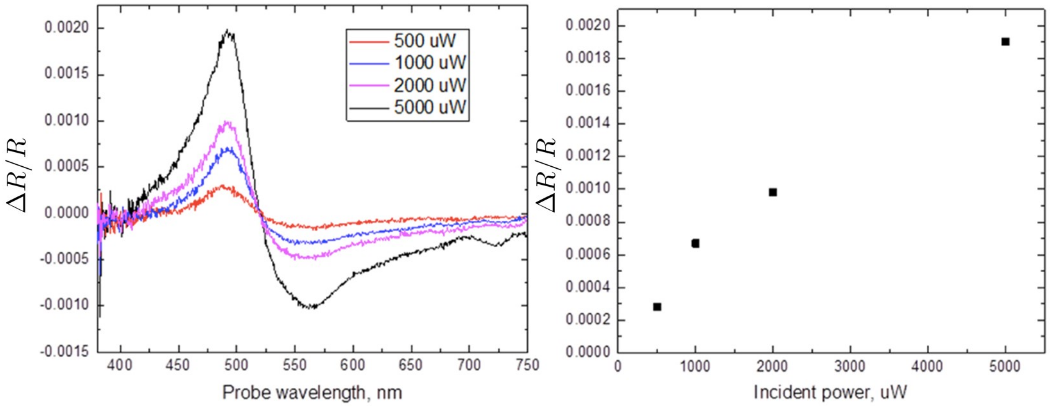

SUPPLEMENTARY FIGURE 5: Power dependence of the experimental differential reflectivity at an early time (122 fs).Full spectrum of the probe reflectivity (left panel) and the maximum of the reflectivity peak(right panel).

We take [9] $\gamma = 70$ J m^{-3} K^{-2}, and $C_l = 2.5$ x 10^6 J m^{-3} K^{-1}. The full-width at half-maximum of the laser pulse is $t_{pulse} = 130$ fs in our case and B is determined to be such that the time integral of $I(t)$ is the fluence, which we have estimated to be 640 μJ/cm^2 = 6.4 J/m^2 for our experiments. We focus on the case of normal incidence case with incident light having wavelength 1200 nm. Fresnel calculations then suggest that $A = 0.03$ and $\alpha^{-1} = 11.8$ nm. We take $a = 1/\tau_{\rm th}$ and $b = 1/\tau_{\rm p}$ where $\tau_{\rm th}$ is the electron thermalization time constant and $\tau_{\rm p}$ is the non-thermal electron-phonon relaxation time constant. We employ $\tau_{\rm th} = 100$ fs, $\tau_{\rm p} = 1.4$ ps, and $g = 2$ x 10^{16} W m^{-3}. These parameter values are typically used for gold, except for the value of $\tau_{\rm th}$, which is smaller than the 500 fs value of Ref. 6 which is typically assumed. Note, however, that in general the early times for which $\tau_{\rm th}$ is relevant pertain to times for which our Δf functions will contain a significant amount of non-thermal contributions and so we probably cannot reliably infer this parameter from our data.

Supplementary Figure 4 displays the resulting electron temperature $T_{\rm e}$ dynamics (green curve). We compare these extended two-temperature results to electron temperatures determined from fits of the normalized $\Delta f(E)/\Delta f_{\rm max}$ electron population changes inferred from the experimental data, where $\Delta f(E) = 1/(\exp(E/kT_{\rm e})+1) - 1/(\exp(E/kT_0)+1)$ and $\Delta f_{\rm max}$ is the maximum value of this difference. This result is shown as the purple curve in Supplementary Figure 4. While the present effective two-temperature model underestimates the maximum temperature, it is seen to be a reasonable description of the results. It is possible to much achieve better agreement if the fluence were increased and/or the absorption coefficient were increased, reflecting more energy being pumped into the sample than expected on the basis of our limiting formulae.

An interesting issue arises concerning radiation penetration depths that should be noted. It turns out that the skin-depth for gold (i.e., its inverse absorption coefficient) for wavelengths near the pulse wavelength of 1200 nm is on the order of 10nm and that the skin-depth for shorter wavelengths (e.g., 520 nm) consistent with the probe light is on the order of 25nm. This discrepancy in skin depths can have consequences for gold films that are $d = 30$ nm or larger because the non-thermal electron disturbance created by the pump corresponds to just a portion of the film thickness whereas the probes are sampling the entire film thickness. If $s_{\rm pump}$ is the skin depth associated with the pump, and $s_{\rm probe}$ is the skin-depth associated with the probe, then a correction factor of $s_{\rm probe}(\omega)/s_{\rm pump}$ should be applied to the $\Delta\epsilon''$ values inferred from the probe-based reflection measurements. We have not investigated this correction but anticipate that it could lead to somewhat larger magnitude $\Delta f(E)$ values.

SUPPLEMENTARY NOTE 9. POWER DEPENDENCE

First, we present the differential reflectivity data at early times (122 fs) for normal incidence for four different excitation powers ranging from 500 μW to 5 mW, Supplementary Figure 5. From this figure it is clear that the dependence of the pump-probe response is close to linear. The left panel shows the full spectrum of the probe reflectivity and the right panel shows the maximum of the reflectivity peak as a function of pump power. The linear dependence on the pump power would imply the absence of multi-photon processes. To examine the power dependence in more detail we plot in Supplementary Figure 6 the normalized differential reflectivity spectra for two different times

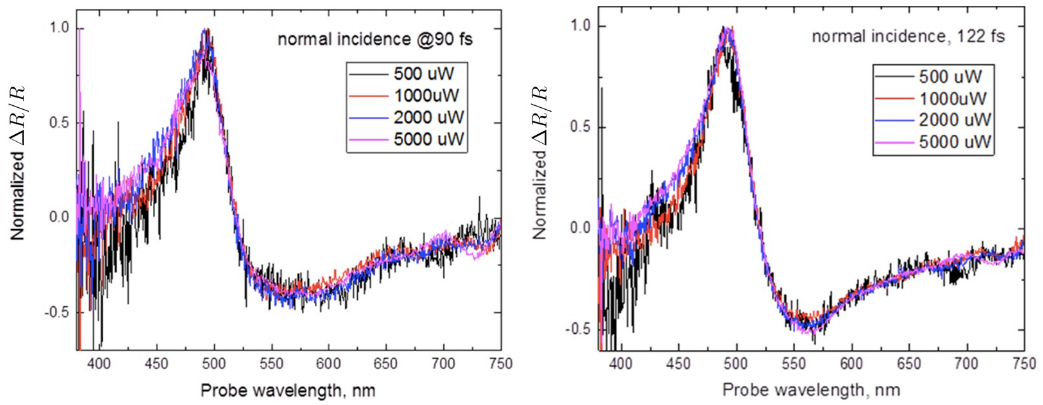

SUPPLEMENTARY FIGURE 6: Power dependence of the experimental differential reflectivity at two different times. The curves are normalized to have the same maximum.

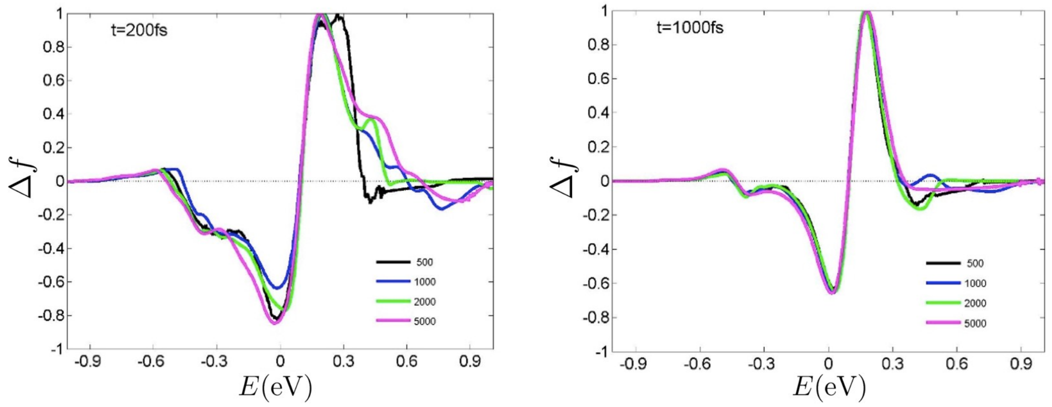

SUPPLEMENTARY FIGURE 7: Calculated population changes, Δf, inferred from our double-inversion procedure, at early and late times for various powers. The curves are normalized to have the same maximum

(90 fs and 122 fs). The normalized spectra nearly collapse into a single curve indicating that one-photon processes dominate the population change across the whole measured spectrum. This not very surprising given the fact that we are probing only < 1 eV above and below the Fermi level whereas the pump photon energy is 1 eV. The only noticeable deviation in the normalized spectra is the short wavelength (high energy) tail of the spectra where we see slight broadening of the signal at higher power. The deviation is small indicating that the contribution of multi-photon processes is negligible.

To quantify this non-linear optical contribution we have calculated the carrier population change (Δf) using our double inversion method, Supplementary Figure 7. The population change is calculated for two different times: early time when the electron gas is not completely thermalized (left panel) and at later time when it is fully thermalized. As it can be seen from these normalized curves the dependence of the width of these curves on the excitation power is very small (it is nonexistent for thermalized gas). Most importantly the observed difference is much smaller than the wide distribution observed for Kretschmann configuration shown in Fig. 6 in the main text. Given that in Kretschmann configuration (Fig. 6) $\Delta R/R$ signal strength is comparable to the signal strength for normal incidence at 5 mW (11 mOD vs 9 mOD) we can conclude that the observed broad distributions for Kretschmann configuration in Fig. 6 are very unlikely to be caused by multi-photon processes. Thus qualitatively different mechanism (such as Landau damping) is needed to explain the broad distribution of Δf in Fig. 6.

SUPPLEMENTARY REFERENCES

[1] Rosei, R., Antonangeli, F. & Grassano, U. d bands position and width in gold from very low temperature thermomodulation measurements. *Surface Science* **37**, 689–699 (1973).

[2] Guerrisi, M., Rosei, R. & Winsemius, P. Splitting of the interband absorption edge in au. *Physical Review B* **12**, 557 (1975).
[3] Johnson, P. B. & Christy, R. W. Optical constants of the noble metals. *Physical Review B* **6**, 4370–4379 (1972).
[4] Christensen, N. E. & Seraphin, B. Relativistic band calculation and the optical properties of gold. *Physical Review B* **4**, 3321 (1971).
[5] Hansen, P. C. Analysis of discrete ill-posed problems by means of the l-curve. *SIAM review* **34**, 561–580 (1992).
[6] Sun, C.-K., Vallée, F., Acioli, L. H., Ippen, E. P. & Fujimoto, J. G. Femtosecond-tunable measurement of electron thermalization in gold. *Phys. Rev. B* **50**, 15337–15348 (1994).
[7] Carpene, E. Ultrafast laser irradiation of metals: Beyond the two-temperature model. *Phys. Rev. B* **74**, 024301 (2006).
[8] Della Valle, G., Conforti, M., Longhi, S., Cerullo, G. & Brida, D. Real-time optical mapping of the dynamics of nonthermal electrons in thin gold films. *Phys. Rev. B* **86**, 155139 (2012).
[9] Conforti, M. & Della Valle, G. Derivation of third-order nonlinear susceptibility of thin metal films as a delayed optical response. *Physical Review B* **85**, 245423 (2012).
